# ALL-1/MLL1, a homologue of Drosophila TRITHORAX, modifies chromatin and is directly involved in infant acute leukaemia

**DOI:** 10.1038/sj.bjc.6601639

**Published:** 2004-02-17

**Authors:** E Canaani, T Nakamura, T Rozovskaia, S T Smith, T Mori, C M Croce, A Mazo

**Affiliations:** 1 Department of Molecular Cell Biology, Weizmann Institute of Science, Rehovot, Israel 76100; 2 Kimmel Cancer Center, Thomas Jefferson University, Philadelphia, PA 19107, USA

**Keywords:** acute lymphoblastic and myelogeneous leukaemia, mouse models, chromatin, epigenetic marking

## Abstract

Rearrangements of the *ALL-1/MLL1* gene underlie the majority of infant acute leukaemias, as well as of therapy-related leukaemias developing in cancer patients treated with inhibitors of topoisomerase II, such as VP16 and doxorubicin. The rearrangements fuse *ALL-1* to any of >50 partner genes or to itself. Here, we describe the unique features of *ALL-1*-associated leukaemias, and recent progress in understanding molecular mechanisms involved in the activity of the ALL-1 protein and of its Drosophila homologue TRITHORAX.

## ACUTE LEUKAEMIAS ASSOCIATED WITH *ALL-1* REARRANGEMENTS

Chromosome band 11q23 is involved in reciprocal chromosome translocations in 5–10% of children and adults with acute lymphoblastic leukaemia (ALL) or acute myelogeneous leukaemia (AML). Nearly all these rearrangements affect the *ALL-1* gene, also termed *MLL1*, *HRX* and *HTRX*, located at 11q23. The translocations occur within an 8.3 kb breakpoint cluster region of the gene and result in the replacement of *ALL-1*-coding sequence 3' to the breakpoint by the coding sequence of the translocation partner (Gu *et al*, 1992; Tkachuk *et al*, 1992). In a second and less frequent type of rearrangement, *ALL-1* undergoes partial tandem duplication (self-fusion), resulting in the production of a larger protein (Schichman *et al*, 1995). The most common *ALL-1* translocations are t(4; 11), t(9; 11), t(11; 19), t(6; 11) and t(10; 11), accounting for 40, 27, 12, 5 and 5% of cases, respectively. However, the total number of different loci participating in *ALL-1* translocations is amazingly high and has already exceeded 50 (Huret *et al*, 2001).

There is an association between particular chromosomal translocations involving *ALL-1* and subtypes of leukaemias. Thus, t(4; 11) nearly always occurs in ALL, t(9; 11) is mostly associated with AML, as are t(11; 19)(q23;p13.1), t(6;11), t(10;11) and the partial duplications. t(11; 19)(q23;p13.3) occurs in both AML and ALL. *ALL1*-associated ALLs are characterised as CD10^−^, CD19^+^ and by B-cell precursor phenotype (pro B), with a high frequency of myeloid-associated (CD15 and/or CD65) antigen expression. Hence, they are also designated biphenotypic or mixed-lineage leukaemia. The AMLs are usually of the myelomonocytic (FAB-M4) or monoblastic (FAB-M5) morphological subtypes and sometimes express cell-surface lymphoid markers. The prognosis of patients with 11q23 abnormalities is dismal. Age is an important prognostic factor. Thus, less than 25% of infants (<1 year) and adults older than 40 or 50 years, with t(4; 11) or t(9; 11), are cured. Patients of intermediate age, in particular 2–9 or 1–14 years old, have significantly better prognosis (Johansson *et al*, 1998; Swansbury *et al*, 1998; Pui *et al*, 2002).

The hallmark of *ALL1*-associated leukaemias is their epidemiology (reviewed in Greaves, 1999; Biondi *et al*, 2000). They predominate infant acute leukaemia (80% of ALL and 65% of AML), and account for the majority of therapy-related (secondary) leukaemias developing in 5–15% of primary cancer patients who had received intensive chemotherapy, including epipodophyllotoxins (VP16) and anthracyclines (doxorubicin) (Pui and Relling, 2000). These drugs are inhibitors of topoisomerase II, and act by stabilising double-strand DNA breaks generated by this enzyme. In both infant and secondary leukaemias, the latency period is remarkably brief. In infants, *ALL-1* rearrangements can be already detected *in utero* and the average age at diagnosis is 6 months, and in secondary leukaemias, latency averages around 18–30 months (reviewed in Greaves, 1999; Pui and Relling, 2000). The very brief time between the initiating event and clinical disease, in conjunction with the high rate of concordance in identical twins with infant leukaemia, points to the effectiveness of ALL-1 fusion proteins in driving the initial clone into frank leukaemia. Based on the findings from therapy-related leukaemia, it was hypothesised (Ross *et al*, 1994) that transplacental exposure of the foetus to natural substances that inhibit topoisomerase II (such as flavonoids in foods and drinks) might play a role in infant leukaemia. Such exposure will induce accumulation of DNA's free ends from within the breakpoint cluster region of *ALL-1*, and this would eventually result in 11q23 translocations. The results of recent biochemical experiments (Strick *et al*, 2000) are consistent with this hypothesis. A structural feature shared by many topoisomerase II inhibitors, including anticancer drugs, is the quinone moiety. The metabolism of quinones is controlled by the enzyme NQO1 that converts toxic benzoquinones to hydroxyquinones. A polymorphism in nucleotide 609 of the enzyme substitutes a proline into serine and consequently inactivates the enzyme. Comparison of paediatric leukaemias with and without ALL-1 rearrangement indicated significant bias in the former for NQO1 genotypes conferring low or no enzymatic activity (Wiemels *et al*, 1999; Smith *et al*, 2002), suggesting that inactivating NQO1 polymorphism increases the risk for ALL-1-associated leukaemias.

## MOUSE MODELS OF *ALL-1* LEUKAEMOGENESIS

Two methodologies have been used to generate mouse models of *ALL-1* leukaemogenesis. Knockin of the partner gene *AF9* into the *ALL-1* locus by homologous recombination resulted in animals that developed AML at approximately 6 months (Dobson *et al*, 1999). Transmission of the transgene in the germ line and its expression during embryogenesis resulted in viable progeny. This, together with the observation that mice carrying a single null allele of *ALL-1* were not predisposed to AML (Corral *et al*, 1996), rules out a model of leukaemogenesis due to haploinsufficiency (passive loss of function). Rather, an extensive series of investigations applying retro viral transduction into cultured haematopoietic progenitor cells, followed by transplantation into mice, have established that *ALL-1* fusion genes act as dominant alleles and the disease is due to *ALL-1* gain of function. Structure–function analysis of several ALL-1 fusion proteins showed that particular elements within the partner proteins, such as transcription effector domains and protein–protein interaction motifs, are critical for the leukaemogenic activity (reviewed in Ayton and Cleary, 2001). The presence of dimerisation domains in some of the partner proteins, such as AF6, AF10, AF17, AF1p, GAS7 and AF3p21 suggested that self-association might underlie the role of the partner proteins with such motifs in the leukaemogenicity of ALL-1 fusion proteins. The most compelling experimental evidence for this is that homodimerisation of ALL-1 by synthetic dimerisation modules mimics many of the features of ALL-1 fusion proteins (Dobson *et al*, 2000; So *et al*, 2003b, Martin *et al*, 2003). Also, disruption of the dimerisation motifs in some partner proteins eliminated pathogenicity. Although self-dimerisation appears to play a key role in leukaemogenesis associated with partner proteins containing a dimerisation motif, the most common fusions–ALL-1/AF4, ALL-1/AF9 and ALL-1/ENL–do not dimerise.

Both the knockin and retroviral transduction approaches turned out to be biased towards myeloid leukaemia. However, very recent studies with virally transduced ALL1-ENL (Zeisig *et al*, 2003) and ALL1-GAS7 (So *et al*, 2003a) showed transformation of biphenotypic lymphoid/myeloid cells and induction of biphenotypic leukaemia. So *et al* serially plated the transduced bone marrows in methylcellulose and found that the fusion protein conferred long-term growth capacity to multipotential progenitors. The progenitors spontaneously differentiated into biphenotypic and myeloid progenitors during culturing *in vitro*. Moreover, mice injected with the transduced serially-plated progenitors developed either biphenotypic leukaemia, or AML or ALL. On the basis of these experiments, it was proposed that ALL-1 fusion proteins enhance the self-renewal potential of multipotent progenitors that retain an ability to differentiate into downstream progeny. The latter are more susceptible to differentiation blocking by ALL-1 fusion proteins, or are prone to secondary mutations required for full malignant transformation.

## GENE EXPRESSION PROFILES OF *ALL-1* LEUKAEMIAS

DNA microarrays technology has been recently applied to construct expression profiles of ALLs and/or AMLs with *ALL-1* rearrangements (Rozovskaia *et al*, 2001, 2003; Armstrong *et al*, 2002; Yeoh *et al*, 2002). Both *ALL-1*-associated ALLs and AMLs have distinct expression patterns that distinguish them from other types of ALL and AML, respectively. The distinction is more robust for ALL, probably reflecting the unusual clinical and biological features and the biphenotypic trait of the disease, not shared by other classes of ALL. In one study (Rozovskaia *et al*, 2003), genes whose expression pattern separated ALLs with t(4;11) from other ALLs were further subdivided, enabling the identification of two subfamilies of t(4;11) tumours. In that study, a substantial number of the genes, which were found to correlate in their expression with the t(4;11) genotype, had been previously associated with cancer, apoptosis or growth control. These included overexpressed oncogenes (such as *HOX A9*, *HOX A10*, *MEIS1*, *LMO2* and *MYC*), overexpressed genes involved in protection from apoptosis and in survival, underexpressed proapoptotic genes, overexpressed genes involved in drug resistance and underexpressed tumour suppressors and growth inhibitors such as *FHIT* and *DAPK1*.

Determination of which of the genes pointed out in the DNA microarrays analyses plays a direct role in pathogenesis might come from experiments with mice. One approach will be to alter the expression (e.g. by interference RNA) of a suspected gene in cell lines with 11q23 translocations, and examine whether the capacity of the cell lines to induce leukaemia in mice is lost. The second approach will be to transduce the fusion genes into mice null for genes suspected of playing an essential role in *ALL-1* pathogenesis, and examine the effect on leukaemia development. Such an experiment has been recently reported for *HOX A9* and *HOX A7* (Ayton and Cleary, 2003). These two genes were found required for *in vitro* myeloid immortalisation by the MLL/ENL fusion protein transduced by a retroviral vector into bone marrow cells. Further, *HOX A9* was found to be indispensable for ALL-1-dependent leukaemogenesis *in vivo*. Applying a complementary approach, Kumar *et al* (2004) bred mice to obtain animals transgenic for *MLL-AF9* and null for *HOX A9* and examined leukaemia development. Surprisingly, the absence of *HOX A9* did not affect the incidence and latency of leukaemia, although the malignant cells displayed a more immature myeloid phenotype. The authors suggest that several *HOX* genes might have to be inactivated to impair leukaemogenicity. The reasons for the contrasting conclusion obtained by the two approaches are currently not known.

## BIOLOGICAL AND BIOCHEMICAL ACTIVITY OF ALL-1 AND ITS DROSOPHILA HOMOLOGUE TRITHORAX (TRX)

*ALL-1* and *TRX* are members of the evolutionary conserved gene family termed as the Trx group (TrxG) that are positive regulators of the homeotic gene complex (*HOX*) during development, and whose activity is opposed by the repressive activity of the Polycomb group (PcG). The TrxG and PcG proteins are not required for initiation of *HOX* genes activity, but act to maintain transcriptional states through later stages of development (reviewed in Brock and van Lohuizen, 2001; Simon and Tamkun, 2002). *ALL-1* also acts at the stage of maintenance (Yu *et al*, 1998). Since considerably more genetic and biological information is available about *TRX*, in comparison to *ALL-1*, here we will emphasise studies of the former. Approximately 20 genes have been characterised as members of the fly TrxG; however, this number is rapidly growing based on genetic and molecular criteria (Gildea *et al*, 2000; Faucheux *et al*, 2003). Further, it has became apparent that the target genes of the TrxG are not limited to *HOX* genes (Kuzin *et al*, 1994; Maurange and Paro, 2002; Beltran *et al*, 2003). In this context, we have recently demonstrated (Smith *et al*, 2004) that following heat induction, the TRX complex is quickly recruited to several heat-shock genes on salivary polytene chromosomes, where it is required to maintain a high level of already initiated transcription. Such a role is similar to the role of TRX in *HOX* gene expression.

Much effort was devoted to *in vivo* analysis of the properties of TrxG and PcG response elements (TRE and PRE, respectively) at target loci in Drosophila. The emerging picture of the best studied target, the regulatory region of the Drosophila *Bithorax* complex *BX-C* gene *Ubx*, suggests that although TREs and PREs are intermingled within a 1.5 kb region *bxd*, they may represent separable regulatory elements (Tillib *et al*, 1999). It is still not clear whether the TrxG and PcG proteins occupy their respective responsive elements at all time, or only when the target gene is active or repressed, respectively. Evidence that these proteins may occupy their binding sites depending on the status of gene expression came recently from analysis of Drosophila larval fat body (Marchetti *et al*, 2003). *BX-C* genes are repressed in the anterior region of the fat body, but are active in the mid-posterior part. In parallel, the PC protein (but not TRX) was detected at the cytological site of bithorax on polytene chromosomes from the anterior part of the fat body, while a strong signal of TRX (but not of PC) was detected on chromosomes from the mid-posterior fat body.

The mechanism by which TRX and ALL-1 reach their target loci is not known. The absence of sequence-specific DNA-binding protein among components of the TRX and ALL-1 complexes (see later) emphasises this quandary. Several recent studies (Bender and Fitzgerald, 2002; Hogga and Karch, 2002; Rank *et al*, 2002) showed that ectopic or induced transcription through regulatory elements of the *Bithorax* complex switches the elements from silenced to activated states. On the basis of this, it was proposed that passage of the RNA polymerase II complex through a regulatory element displaces the repressive PcG complex, set there by default, and enables recruitment of the TRX complex that imprints epigenetic marks instructing transcription of the adjacent *HOX* gene.

Chromatin immunoprecipitation (ChIP) experiments previously identified TRX both on TREs and on promoters within the *Bithorax* gene complex (Orlando *et al*, 1998). The ChIP methodology was recently applied to ALL-1 (Nakamura *et al*, 2002; Milne *et al*, 2002) and the protein was found bound at the promoters of *HOX A9* and *HOX C8* in cultured cells expressing these genes (homologues of Drosophila TREs and PREs have not been identified yet in mammals). Elimination of ALL-1, by applying interference RNA methodology, blocked transcription of *HOX A9*. Both studies showed that ALL-1 is a histone methyltransferase that methylates lysine 4 of histone H3. The enzymatic activity is conferred by the C-terminal SET domain. The presence of ALL-1 on the promoters of the two *HOX* genes is linked to H3-Lys4 methylation as well as to acetylation of histones H3 and H4 at the promoter. Several other TrxG, PcG, and other chromatin – associated proteins containing SET domains – were recently shown to methylate histones at specific residues (reviewed in Orlando, 2003). Such marking of histones (as are acetylation, phosphorylation and ubiquitination of specific histone residues) conveys heritable transcription patterns (expression or silencing) in an epigenetic manner.

The molecular mechanisms by which TRX and ALL-1 act was further elucidated by the identification and characterisation of stable protein complexes associated with the two proteins. TRX is a component of the TAC1 complex that includes the histone acetyltransferase (HAT) dCBP, and a SET - binding factor, SBF1 (Petruk *et al*, 2001). The recruitment of TAC1 components to the *HSP70* locus following heat induction results in enhanced expression of the gene and correlates with acetylation and methylation of histones (Smith *et al*, 2004). The ALL-1 complex (Nakamura *et al*, 2002) completely varies from that of TRX. It contains at least 29 proteins. The majority of the latter are components of seven subcomplexes involved in transcription preinitiation, nucleosome remodelling, histone deacetylation, histone methylation or RNA processing. The purified ALL-1 complex methylates, acetylates, deacetylates and remodels nucleosomes and is bound at the promoter of the target gene *HOX A9*. It appears that a major role of ALL-1 is to assemble this large supercomplex of enzymatic activities. In summary, the recent biochemical experiments indicate that TRX and ALL-1 recruit to target genes a host of enzymatic activities, mostly involved in epigenetic marking. The way in which these marks are inherited to progeny cells (memorized) is not known (they have to survive DNA replication, during which the histones are thought to be stripped off the DNA), but it has been speculated (Turner, 2000; Richards and Elgin, 2002) that the modified histones stay at or near the replication fork and are incorporated into the daughter DNA strands.

## UNRESOLVED ISSUES AND FUTURE DIRECTIONS

While considerable progress was made in identification of the biochemical activities of the ALL-1 and TRX protein complexes, the mechanism by which these complexes are recruited to specific sequences (genes) is completely unknown. The recent reports that transcription of regulatory regions induces heritable transcriptional activation of the Drosophila *BX-C* genes raises the possibility that such a mechanism is involved in the recruitment of TRX and ALL-1 complexes. Another outstanding issue is the identification of genes, other than *HOX*, that are regulated by ALL-1 and TRX. The demonstration that the mammalian PcG protein BMI-1 regulates the important cell cycle locus *INK4a/ARF* (Jacobs *et al*, 1999) suggests that looking for additional ALL-1/TRX targets (e.g. by conditional elimination or activation of the two proteins, followed by DNA microarray analysis) might be rewarding. Further, the process by which the ALL-1-mediated histone H3-Lys4 methylation induces chromatin alteration (presumably decondensation) and gene expression, and the precise roles of the transcriptional subcomplexes within ALL-1 supercomplex, have yet to be determined. A new line of investigation is likely to originate from the recent remarkable finding that the PHD finger motif of the ING2 protein binds phosphoinositides, and that this interaction regulates the biological activity of the latter (Gozani *et al*, 2003). The presence of four PHD finger motifs in both ALL-1 and TRX points to a potential central mechanism by which these proteins are regulated. Finally, the reason why ALL-1 (and presumably TRX) is proteolytically cleaved by an evolutionary conserved enzyme into two polypeptides, which are held together, is not known (Nakamura *et al*, 2002; Yokoyama *et al*, 2002; Hsieh *et al*, 2003).

Mouse models have recently identified the type of precursor cell transformed by ALL-1 fusion proteins, and DNA microarrays analyses focused attention on genes that might play a direct role in leukaemogenesis. Identification of such genes is clearly a focal point for understanding the disease in molecular terms and for future therapeutic intervention. However, other central questions remain. First, do all ALL-1 fusions, including self-fusions, trigger a single pathway leading to disease? Why are most fusions associated with specific leukaemia subtype (e.g. ALL1-AF4 with ALL, ALL1-AF6 with AML)? Second, since the fusion proteins lack the SET domain with its histone H3-Lys4 methylation activity, how do they retain transcriptional activation capacity? Do they target all genes regulated by normal ALL-1? Do they compete with normal ALL-1 on the same targets? Do they block transcription of some loci? Also, the absence of the PHD fingers in ALL-1 fusion proteins should relieve them from potential regulation by phosphoinositides; whether this is an important aspect of the leukaemogenic activity of these proteins has yet to be determined. A third issue is whether ALL-1 fusions confer resistance to apoptosis. ALL-1-associated leukaemias are notorious for their poor prognosis with chemotherapy, and exhibit drug resistance. Since it is now believed that the cytotoxic action of most chemotherapeutic drugs is through activation of apoptotic pathways, drug resistance of ALL-1 leukaemias might be due to disruption of apoptotic processes. Such disruptions appear to be crucial for the development of many tumours (reviewed in Johnstone *et al*, 2002). Still, very little has been reported about this issue with regard to ALL-1 leukaemias. In one study (Kersey *et al*, 1998), ALLs with t(4; 11) were compared to similar tumours without t(4; 11) and found to be dramatically more resistant to serum deprivation stress. A recent investigation (Wiederschain *et al*, 2003) showed nuclear colocalisation of the fusion protein ALL1-ELL with p53, and loss of p53-mediated apoptosis. Whether this phenomenon is unique to that particular fusion protein or is it of a more general nature is presently not known.
